# A Review of Functions of Speculative Thinking

**DOI:** 10.3389/fpsyg.2021.728946

**Published:** 2021-10-15

**Authors:** Lun Huang, Yibo Xie, Xiaolin Chen

**Affiliations:** Management School, Hainan University, Haikou, China

**Keywords:** speculative thinking, counterfactual thinking, prefactual thinking, functional theory, preparatory function

## Abstract

Speculative thinking refers to thinking about past or future possibilities; it includes counterfactual thinking, prefactual thinking, and other types. In this narrative review, we discuss the traditional function of speculative thinking in improving future performance (i.e., the preparatory function). We also explore several non-preparatory functions of speculative thinking that have not been widely covered, namely the functions of conveying information and of supporting lying. In addition, we address temporal asymmetry; one perspective focuses on psychological distance in speculative thinking about the past and future, while another focuses on temporal asymmetry and reality/hypothetical differences in the preparatory function of speculative thinking. Overall, this review suggests that a broader functional theory is needed to address non-preparatory functions and the traditional preparatory function. Such a theory should cover all speculative thinking about the past and future rather than simply counterfactual thinking.

## Introduction

In daily life, people often consider how things could have been different in the past and might be different in the future. Imagine, for example, the following scenario: you rush to the train station, only to discover that your train departed 5 min ago. You may think about how things could have gone differently, such as “If I hadn’t gotten caught in that traffic jam, I would have arrived at the train station on time.” You may also think about how to ensure a different outcome in the future: “If I leave home earlier next time, I’ll arrive at the train station on time.” Imagining how events could have been different is called *counterfactual thinking* (e.g., Kahneman and Tversky, 1981; Roese, 1997; Byrne, 2002). Kahneman and Tversky (1981) proposed this concept in a paper on heuristic simulation. They described heuristic simulation as a conscious reactivation of past behavior stored in memory. Imagining how things might or will differ in the future represents *prefactual thinking* (e.g., Schacter et al., 2007; Epstude et al., 2016). Counterfactual and prefactual thinking are two types of speculative thinking; they involve consideration of past or future possibilities, respectively.

This narrative review explores the functions of speculative thinking about the past and future from a general perspective. We also focus on the traditional function of speculative thinking (i.e., improving future performance) as well as several functions that have not been widely discussed (i.e., the function to convey information through others’ counterfactuals and the function of generating lies). We further investigate different functions in speculative thinking about the past and future. First, we review the literature on thinking about the past and future along with research on counterfactual thinking (i.e., speculative thinking about the past).

### Thinking About the Past and Future

Differences apply when thinking about the past and future – past events actually occurred, whereas future events are based on predictions or judgments. Anderson and Dewhurst (2009) found that past events contain more perceptual details than future events. In their study, adult participants were asked to either imagine future events or recall past events. Participants in the future group completed the sentence “Next year I,” and participants in the past group completed the sentence “Last year I.” Participants in the future group mentioned more general events (e.g., “Next year I will have the best summer”), whereas those in the past group cited more specific events (“Last year I took my driving test”). These results suggest that when individuals think about the past, they can more easily access direct experiences based on events. Therefore, pondering the past (vs. speculating about the future) will lead to clearer representations of temporal and spatial information along with more coherent storylines.

The difference between thinking about the past and future is further reflected in people’s tendency to imagine future events (compared to recalling past events) as positive. An optimistic bias is thus common when thinking about the future (e.g., Taylor and Brown, 1988; D’Argembeau and Van der Linden, 2006). For instance, D’Argembeau and Van der Linden (2006) explored individual differences in evaluating the valence of past and future events by asking participants to either remember past events (e.g., yesterday, a week ago, a month ago) or imagine future events (e.g., tomorrow, in a week, in 1 month). Participants were instructed to write a brief description of each event and then evaluate its valence on a 7-point scale (“When/if this event happened, my emotions were/would be: −3 = *very negative*, 0 = *neutral*, +3 = *very positive*”). Findings showed that participants usually perceived future events more positively than past events.

Scholars have also conducted neuroimaging studies to investigate differences when considering the past and future. Addis et al. (2007) explored brain region activation when people think about the past and future. The authors asked participants to imagine an event occurring within a specific period (a week, a year, 5–20 years) in the past or in the future. Results indicated that the left hippocampus and posterior visuospatial regions are often both engaged in past and future event thinking. However, thinking about the future leads to activation of the right hippocampus as well. Other researchers have suggested that thinking about the future (vs. the past) produces greater activation of the posterior parietal cortex, which plays a role in attention. Thinking about the future therefore appears to require more attentional resources than thinking about the past (e.g., Cabeza, 2008; Ciaramelli et al., 2008).

In this section, we reviewed research related to comparing thinking about the past and future along with the literature on general speculative thinking about the past and future. Counterfactual thinking and prefactual thinking are two typical forms of speculative thinking, although other types also exist. Generally, speculative thinking involves past or future possibilities.

### Psychological Distance of Speculative Thinking

When people plan for the future, they can consider a distant future (e.g., in 1 year) or a near future (e.g., tomorrow). They can also recall an event in the distant past (e.g., last year) or in the near past (e.g., yesterday). Liberman et al. (2007) suggested that future temporal distance is related to people’s savings, purchases of items intended for future use, and actions to achieve goals (e.g., self-control). Past events may evoke regret. Temporal distance is an aspect of psychological distance (e.g., Trope and Liberman, 2003, 2010). Construal level theory (CLT) (e.g., Trope and Liberman, 2010) entails how different types of psychological distance influence people’s representations of events. In particular, farther psychological distance can engender more abstract thinking, whereas nearer psychological distance can result in more specific thinking.

In this review, *speculative thinking* encompasses thoughts about past or future possibilities. Thinking about events in the past and future with different psychological distances hence falls under speculative thinking. Scholars have suggested that thinking about close psychological distance may be more helpful to future behavior than thinking about far psychological distance. In one case, Peetz et al. (2009) explored the impact of psychological distance on the motivation behind individuals’ academic performance. Participants in the close condition marked the distance between “today” and “graduation” on a timeline spanning 25 years, while those in the distant condition marked a 5-year timeline. Because both timelines were of the same length, points on the 25-year scale were closer than those on the 5-year scale. This manipulation enabled participants to think that the longer timeline (vs. that spanning 5 years) involved closer psychological distance between graduation and now. Participants then evaluated their motivation for academic performance. Compared with participants given a 5-year timeline (i.e., who were primed to think about far psychological distance), those given the 25-year timeline (i.e., close psychological distance) were more motivated to improve their academic performance.

### Counterfactual Thinking

Counterfactual thinking represents the main research area related to speculative thinking, as relatively fewer studies have explored other kinds of speculative thinking (e.g., prefactual thinking). Studies and theories on counterfactual thinking can offer a theoretical backdrop to explore general speculative thinking. We address this form of thinking (as a specific form of speculative thinking about the past) in the following section.

#### Classifications of Counterfactual Thinking

Roese and Olson (1993) identified three types of counterfactual thinking: additive, subtractive, and substitutive. Additive counterfactual thinking includes some imaginary antecedents, subtractive counterfactual thinking removes some imaginary antecedents, and substitutive counterfactual thinking replaces original antecedents with alternative (imaginary) antecedents and reconstructs an event. For example, a student could think counterfactually that “If I had come to the review session, I could have passed the exam this time,” this is an additive counterfactual statement because “come to the review session” did not actually happen. The sentence “If we did not have a drink, we would have caught the train” is a subtractive counterfactual statement. The sentence “If I studied instead of playing video games, I would have passed this exam” is a substitutive counterfactual statement. Markman et al. (1993) classified counterfactual thinking into upward and downward forms. Upward counterfactual thinking focuses on how prior outcomes could have been better (e.g., an athlete thinking “If we had practiced more, we could have won the match”), whereas downward counterfactual thinking focuses on how the outcomes could have been worse (e.g., “We would have lost the game if we hadn’t practiced”).

#### Determinants of Counterfactual Thinking

Early research on counterfactual thinking investigated the determinants leading to such thinking (e.g., Kahneman and Miller, 1986). For example, Kahneman and Tversky (1981) found that an exceptional, unanticipated outcome is more likely to cause people to think counterfactually than a normal outcome that aligns with one’s expectations. In one experiment, researchers asked participants to read an exceptional or unexceptional story and identify that in which the protagonist was more likely to think counterfactually. In the exceptional story, the protagonist took an unusual route when driving home and was involved in an accident; in the unexceptional story, the protagonist chose their usual route home and was involved in an accident. Participants believed that the protagonist in the exceptional story would be more likely to think counterfactually than the protagonist in the unexceptional story.

Studies have also examined the impact of negative outcome valence on counterfactual thinking. Landman (1987) suggested that adverse outcomes tend to lead to counterfactual thinking more than positive outcomes: participants who suffered negative outcomes expressed greater regret and more counterfactual thinking than those who enjoyed positive outcomes. This pattern was thought to manifest because negative outcomes are more likely to be exceptional than positive outcomes (Landman, 1987). Davis et al. (1995) conducted a long-term study on people who had suffered the tragedy of losing their spouse or children in a car accident. Interviews revealed that participants’ reported negative emotions could accurately predict individuals’ frequency of counterfactual thoughts.

Roese (1997) asserted that counterfactual thinking includes an activation stage and a content stage. The activation of counterfactual thinking occurs when a person thinks counterfactually when encountering an event; the content of counterfactual thinking reflects the ideas in people’s counterfactual thoughts. For instance, when a person nearly catches a plane, he may ponder how things might be different next time (i.e., activation of counterfactual thinking). His counterfactual thoughts may be “If I went out earlier, I could have been able to catch the plane” (i.e., content of counterfactual thinking). Roese (1997) noted that the content of counterfactual thinking can vary, but such thinking can only be activated (or not) in the activation stage. Roese (1997) further suggested that counterfactual determinants may affect these stages differently. Typically, outcome valence is the core determinant of counterfactual thinking activation, with exceptionality being the main determinant of counterfactual thinking content.

## Preparatory Function of Speculative Thinking

The functions of counterfactual thinking include the preparatory function and non-preparatory function (Epstude and Roese, 2008; Byrne, 2016). Extensive research has focused on the preparatory function, such as functional theory (Epstude and Roese, 2008). Studies on non-preparatory functions have pertained to the emotional function of counterfactual thinking. For example, upward counterfactual thinking can produce negative emotions while downward counterfactual thinking can generate positive emotions (Roese, 1997). Varied counterfactual thinking can evoke different emotions as well: Niedenthal et al. (1994) found that thinking counterfactually about one’s personality was likely to lead to shame, whereas thinking counterfactually about one’s actual behavior tended to inspire guilt. However, scholars have largely overlooked other non-preparatory functions of counterfactual thinking. Therefore, in this review, we discuss the functions of conveying information and of supporting lying; these non-preparatory functions have rarely appeared in the extant literature.

Most work on the preparatory function of speculative thinking has focused on counterfactual thinking. As an exception, Baumeister et al. (2016) proposed a theory of pragmatic prospection that considers why people think about future possibilities. The authors suggested that thinking about such possibilities can guide subsequent actions to realize desired outcomes. Their theory posits that thinking about future possibilities involves two stages featuring distinct emotional valence. The first stage is optimistic and entails imagining what a person would like to happen. In the second stage, individuals map out how to achieve what they hope will transpire in the future; this stage is characterized by the consideration of obstacles, necessary steps, and other potential problems. It therefore tends toward cautious realism – even pessimism. The two stages jointly serve the preparatory function of future speculative thinking. On one hand, to approach the future, people need to frame future events as positive and meaningful; doing so can provide motivation. On the other hand, preparing for possible obstacles and maintaining defensive pessimism can spark an early adaptive response to potential failure.

### Functional Theory of Counterfactual Thinking

Epstude and Roese (2008) devised the functional theory of counterfactual thinking, which concerns how counterfactual thinking affects subsequent behavior. The functional theory assumes that when people fail to achieve one goal, they will try harder to achieve that goal in the future. This function of counterfactual thinking (i.e., to change future behavior) embodies the preparatory function. Specifically, although counterfactual thinking concerns past events, it helps people prepare for the future. Epstude and Roese (2008) stated that counterfactuals affect future behavior via the content-specific pathway and content-neutral pathway (to be discussed in more depth later in this paper). People who think counterfactually can often infer an outcome’s antecedents, spurring their intention to change and ultimately altering their behavior.

Some researchers have suggested that counterfactual thinking can inspire behavioral regulation due to comparison. Medvec et al. (1995) explored the emotional reactions of silver and bronze medal winners in the 1992 Summer Olympics. They discovered that bronze medal winners were happier than silver medal winners because athletes who won bronze medals compared themselves with players who won no medals. Bronze medal winners’ feelings revolved around how things could have been worse (i.e., “I would have not won a medal if I focused less”), reflecting downward counterfactual thinking. By contrast, silver medal winners compared themselves with gold medal winners. Silver medal winners thus focused on how their outcome could have been better (i.e., “I could have won the gold medal if I used different strategies”), indicating upward counterfactual thinking.

Scholars have yet to explore whether counterfactual thinking unconditionally produces functional outcomes. However, Smallman and Summerville (2018) contended that counterfactual thinking requires four criteria to be met. First, an event’s antecedent can be accurately identified; for example, in the counterfactual statement “If I had not forgotten my umbrella, I would not have gotten wet in the rain,” forgetting an umbrella is explicitly identified as the antecedent of getting wet (rather than some other factor, such as an inaccurate weather forecast). Second, an event’s antecedent is controllable: in the counterfactual statement “If I had not had a cup of coffee, I would not have been late,” the person had control over their decision to drink coffee. Third, counterfactual thoughts can produce specific behavioral intentions or lead to vastly improved motivation. Fourth, a person can execute behavior related to counterfactual thoughts in the future. For instance, for the counterfactual statement “If I had a different exam strategy, I would have done better in the exam,” the student can apply a new strategy to their next test. According to Smallman and Summerville (2018), the degree to which counterfactual thinking generates functional outcomes depends on the extent to which such thinking meets these four criteria.

Notably, Roese and Epstude (2017) presented an updated theoretical synthesis to clarify the functional theory of counterfactual thinking by incorporating recent evidence relevant to this review. Their revision came in response to some studies (e.g., Petrocelli and Harris, 2011) challenging the idea that counterfactual thinking serves a preparatory function and improves actual performance. Similar to Smallman and Summerville’s (2018) suggestion that four conditions must be met for counterfactual thinking to serve a preparatory function, Roese and Epstude (2017) argued that counterfactual thinking does not serve this function unconditionally but instead requires that two steps be met. First, counterfactual thinking must allow people to make accurate causal inferences based on the antecedent that led to the outcome. In Roese and Epstude’s (2017) example of flight simulators, the counterfactual statement “I would have made a better landing if I had remembered to lower my flaps” specifies the antecedent (i.e., lowering flaps) of the outcome (i.e., landing). Conversely, the counterfactual statement “If only I had turned the volume up on my 80 s rock music playlist, I would have made a better landing” does not point out the antecedent. Second, counterfactual thoughts must be applicable to real-life situations, not simply ideas. For instance, in the above example of learning to land an airplane, the trainee can apply the cues from counterfactual thinking to their next flight learning session.

#### Content-Specific Pathway

According to the functional theory, people employ a content-specific pathway to generate particular behavioral intentions from counterfactual thoughts. These intentions can then affect subsequent behavior. For example, if a person gets wet in the rain, then they might think counterfactually that “I could have avoided this if I remembered to bring an umbrella,” in the future, they will bring an umbrella to avoid getting wet. The content-specific pathway implies that, in counterfactual thoughts, information related to the situation can inspire subsequent behavioral intentions, which can then spur changes in future behavior.

Smallman and Roese (2009) used a sequential priming paradigm to study how counterfactual thoughts affect behavioral intention. Specifically, study participants read about a negative event (e.g., milk spilled on the floor). Next, in the counterfactual trial, participants read a counterfactual statement on screen (e.g., “I could have been more careful”); in the control trial, participants were asked to evaluate whether this negative event occurred frequently in their lives. Then, all participants made a behavioral intention judgment by indicating “yes” or “no” to an on-screen statement (e.g., “In the future I will be more careful”). Participants reading counterfactual statements exhibited shorter reaction times for behavioral intention judgments versus participants who did not read such statements. This result suggests that the participants who read counterfactual statements found it easier to generate behavioral intention than those who did not read such statements.

Combining research on the relationship between behavioral motivation and actual behavior can clarify how behavioral motivation following from counterfactual thinking can lead to actual behavior change. Webb and Sheeran (2006) scrutinized the relationship between behavioral intention and actual behavior, although they did not explicitly address counterfactual thinking. In a meta-analysis of 47 experiments, half of all participants were asked to express a behavioral intention about something specific, while the other half expressed no behavioral intention. Webb and Sheeran (2006) found that participants with behavioral intentions were more likely to engage in subsequent behavior than participants without behavioral intentions. Brandstätter et al. (2001) also explored the relationship between behavioral intention and actual behavior. They chose one group of opiate withdrawal participants (i.e., high cognitive load) and one group of post–opiate withdrawal participants (i.e., low cognitive load). All participants were instructed to compose their vitae. Then, half of the participants were told to generate a plan that was unrelated to the main task; the other half generated a plan related to the main task. Even under high cognitive load, participants with specific task-related behavioral intentions were more likely to complete the task (i.e., composing their vitae) than participants with no specific behavioral intentions.

Smallman and McCulloch (2012) investigated speculative thinking about past and future events at different psychological distances. Results showed that temporal distance is a relevant consideration when predicting functional benefits of the content-specific pathway of counterfactual thoughts. Through two experiments, the authors explored the link between psychological distance and counterfactual thinking’s function that can generate behavioral intention. Overall, Smallman and McCulloch (2012) found that thinking counterfactually (i.e., speculative thinking about the past) about close psychological distance was more likely to spark an action-changing plan than thinking counterfactually about far psychological distance. Their two experiments revealed that the preparatory function (i.e., generating future behavioral plans) is sensitive to changes in the temporal distance of related behavioral intention, regardless of whether the temporal distance is in the past or future. Also, compared to behavioral intention in the distant future, counterfactual thinking can better facilitate such intention in the near future. Similar to speculative thinking about the past, the function of counterfactual thinking is influenced by different temporal distances. The function of general speculative thinking may therefore be affected by temporal distance.

Smallman and McCulloch (2012) also highlighted the relevance of elements of construal level theory, suggesting that the content-specific pathway may function most effectively when a match exists between temporal distance and the level of abstraction of relevant behavior. This theory asserts that psychologically close events are more likely to lead to specific action plans than psychologically distant events (e.g., Trope and Liberman, 2010). Trope and Liberman (2010) pointed out that a high construal level corresponds to stable and central features, whereas a low construal level is associated with unstable and superficial features. Therefore, a high construal level can maintain stable event features. Specifically, as the psychological distance increases, high construal–level characteristics (i.e., central features that do not readily change over time) will become increasingly prominent; as psychological distance declines, low construal–level attributes (i.e., superficial features that readily change over time) will become increasingly prominent. Kivetz and Tyler (2007) similarly indicated that a low construal level reflects a realistic state that is easy to change, while a high construal level reflects an idealistic state that is difficult to change.

Distinct construal levels (i.e., abstract or specific) have also been found to affect individuals’ behavior. For example, McCrea et al. (2008) explored the relationship between construal level and people’s intentions to take action. Participants in the low construal level condition described 10 activities using specific language; those in the high construal level condition described the same 10 activities using abstract language. On a scale from 1 (not at all) to 7 (very much), participants then evaluated how convenient it would be for them to complete these activities. Participants in the low construal level condition rated the activities as more convenient than participants in the high construal level condition and reported that they would not postpone completing these activities. A low construal level is thus less likely to cause people to postpone activities than a high construal level.

#### Content-Neutral Pathway

Through the content-specific pathway, information in counterfactual thinking only affects subsequent behavior associated with the specific information. Through the content-neutral pathway, counterfactual thinking can influence people’s motivations and emotions and guide their behavior. Kray et al. (2006) demonstrated that counterfactuals that are unrelated to the task can still lead to better task performance. More specifically, they assigned participants to either a counterfactual or non-counterfactual group who read counterfactual and non-counterfactual stories, respectively. All participants then completed a logical reasoning task that was not related to the stories. Ultimately, the counterfactual group performed better on the logical reasoning task compared with the non-counterfactual group.

Kray and Galinsky (2003) argued that a counterfactual mindset can effectively promote group decision making. Again, participants were assigned to counterfactual or non-counterfactual groups. The counterfactual group read a story about a protagonist who changed her seat number, after which her original seat number was drawn in a lottery (i.e., she could have won the lottery). The non-counterfactual group read a similar story in which the protagonist did not change her seat number and did not win the lottery. After reading this pilot story, which was unrelated to the main study task, all participants imagined they were part of the decision-making team after the Space Shuttle Challenger disaster and they needed to acquire information to make a correct decision. In particular, participants needed to determine the relationship between temperature and the space shuttle’s engine failure and then halt the shuttle’s launch. Participants assigned to the counterfactual condition were more likely to make an accurate decision than those in the non-counterfactual condition. Kray et al. (2006) later suggested that, by identifying and analyzing information that is vital to group decision making, a counterfactual mindset can enhance decision-making accuracy and foster cooperation.

Thinking counterfactually about events allows people to consider how to avoid a negative outcome. Self-efficacy, self-control, and overconfidence evolve throughout this process. For example, when a teacher educates a student, the teacher is not only educating but also sensing that the situation is under control. This feeling of control can be helpful in improving the student’s future education. Nasco and Marsh (1999) explored how counterfactual thinking engenders a sense of control and then leads to better performance. After an exam, participants thought counterfactually about how the exam outcome could have been different. They recalled their counterfactual thoughts 1 month later and were told they would be taking a second exam. Participants then assessed how much control they had over the next exam. Those who engaged in upward counterfactual thinking after the first exam perceived themselves as having greater control over the exam than participants who engaged in downward counterfactual thinking. Perceived control was positively correlated with performance on the second exam. In general, functional theory considers factors such as individuals’ mentality and motivation while stressing the role of counterfactual thinking in behavioral regulation.

In the content-neutral pathway, information obtained through counterfactual thinking affects people’s motivation, which increases their effort toward a goal and thus affects behavior (Smallman and Summerville, 2018). Hammell and Chan (2016) provided evidence of this phenomenon by examining whether counterfactual thinking promotes actual behavioral change (vs. behavioral intention). Participants played video games initially. They then either thought counterfactually or prefactually about their performance or completed an unrelated filler task. Finally, all participants played the video game again. Although the authors found that participants who thought prefactually generated more controllable modifications than participants thinking counterfactually, participants in both the prefactual and counterfactual groups displayed improved task performance compared to participants performing the filler task. The mismatch between the difference in participants’ number of controllable modifications but similarly improved performance suggests that the content-neutral pathway – rather than the content-specific pathway – drove behavioral change. In essence, this outcome implies that even if counterfactual thinking is less likely to focus on controllable modifications, it can still be functional and influence future behavior.

### Temporal Asymmetry in Preparatory Function of Speculative Thinking

The functional theory of counterfactual thinking (Epstude and Roese, 2008) specifically pertains to how counterfactual thinking about what might have been can affect future behavior. The key tenet of this theory is as follows: by imagining a better past, one can prepare for similar future events with the aim of achieving a better outcome. Importantly, however, not all speculative thoughts focus on past events; it is also possible to speculate about the future and to entertain what might happen. Some studies have shown that people think differently about past and future events. Ferrante et al. (2013) discovered that when people think prefactually about future events, they are more likely to consider controllable event aspects compared with thinking counterfactually. These findings challenge the functional theory. For example, Ferrante et al. (2013; Study 1) asked participants to attempt a scrambled word task and then think either counterfactually about their performance or prefactually about a future attempt. Participants who answered the counterfactual thinking question (i.e., “Things would have been better for me if…”) were more likely to focus on the task’s uncontrollable features (e.g., “…if I had longer time”) than those who answered the prefactual thinking question (i.e., “Things will be better for me in the next game if…”). Participants who engaged in prefactual thinking were more apt to focus on controllable features (e.g., “…if I used another strategy”) than those who answered the counterfactual thinking question. Ferrante et al. (2013) thus concluded that prefactual thinking focuses on more controllable premises than counterfactual thinking, such that prefactual thinking is more likely to serve a preparatory function for the future. In sum, types of speculative thinking can vary in how well they prepare people for the future.

### Reality/Hypothetical Difference in Preparatory Function of Speculative Thinking

Speculative thoughts can also differ on another dimension: whether these thoughts concern real or hypothetical events. Whereas some researchers (e.g., Ferrante et al., 2013; Mercier et al., 2017) have framed prefactual thinking in terms of an anticipated future reality, others have construed prefactual thinking as hypothetical. Epstude et al. (2016) suggested that prefactual thoughts take the form “If action X is taken, it will lead to outcome Y,” that is, prefactual thinking concerns how things will vary from the current reality, but without a firm commitment to a specific future event.

Some scholars have suggested that decisions about real events may differ from decisions about hypothetical occurrences (e.g., Galotti, 1989; Galotti et al., 2006; Camerer and Mobbs, 2017). Bostyn et al. (2018) asked one group of participants to imagine that a cage of five mice would receive an electric shock in 20 s, but participants could pull a lever to shift the shock to a cage containing only one mouse. Participants in the other group faced the same dilemma but were led to believe that their decision involved real mice that could be seen in front of them (in reality, no mice were shocked). Participants in the “real” group were less likely to decide to pull the five-mice lever than those in the hypothetical group.

## Lying-Supporting Function of Speculative Thinking

Some studies have explored the link between counterfactual thinking and deception. For instance, Debey et al. (2014) examined whether thinking about the truth forms the basis of lies. They asked participants to answer simple questions (e.g., “Are you a student?”) by indicating “yes” or “no,” but participants were instructed to lie in response to all questions (e.g., students should answer “no” when asked “Are you a student?”). The authors recorded participants’ reaction times and accuracy in answering these questions. Furthermore, when responding to each question, participants were shown random “YES” or “NO” words along with the question being answered. For example, for the question “Are you a student?” (to which students needed to respond “No” to lie and fulfill the experimental requirements), “YES” or “NO” appeared on screen to distract participants’ attention. Researchers measured the reaction time and accuracy of participants’ answers. Results showed that, compared with when the distraction word represented the lie, participants who were presented with questions when the distraction word represented the truth exhibited shorter reaction times and higher accuracy rates for lying. For instance, when answering the question “Are you a student?” participants who saw “YES” gave a “No” answer faster and more accurately than participants who saw “NO.” Debey et al. (2014) therefore proposed that truth activation (i.e., seeing the word that represents the truth) forms the basis of lying (i.e., a briefer reaction time and higher accuracy rate for lying). Briazu et al. (2017) suggested that this heuristic mechanism (i.e., lies are based on truth activation) mirrors counterfactual thinking. More concretely, although counterfactual thinking involves considering information that is not true, such thinking is rooted in what was meant to have happened.

Shalvi et al. (2011) found that seeing a possible alternative outcome to an event can lead to lying. They suggested that this pattern emerges because seeing an alternative outcome allows people to generate counterfactual thoughts. The authors used a “die under cup” task in which participants rolled a die and checked its number in private. In one condition, participants were told that they could only roll the die once and that a larger die number would result in a higher monetary reward. In another condition, participants were told that they could roll the die three times, and the first number rolled determined their reward; the last two rolls were meant to test whether the dice were normal. Shalvi et al. (2011) discovered that rolled numbers reported by participants in the multiple-rolling condition were greater than the normal distribution and greater than in the control condition. The authors presumed that seeing an alternative outcome (which participants could have achieved but did not) led participants to think counterfactually about how they could have rolled a different number. Shalvi et al. (2011) further suggested that counterfactual thinking generated after participants saw the other numbers they rolled (e.g., “I could have rolled a larger number”) might lead them to lie about their rolling number; that is, counterfactual thinking may allow people to justify lying. Observing the desired alternative can therefore reduce the extent to which people believe that lying is unethical, even if such alternatives are not true in reality (e.g., additional rolls did not determine rewards). People use this self-justification to reduce their perception of lying as unethical so they can feel better about being dishonest.

Lelieveld et al. (2016) used a similar die-rolling paradigm to study participants’ neurological responses to evaluating others’ lies about dice results. Results showed that lying about a counterfactual alternative that is closer to the truth is more likely to be considered justifiable than lying about an imaginary alternative that is further from the truth. Briazu et al. (2017) suggested that people lie about the closeness to an expected outcome because individuals believe it is acceptable for themselves and others to do so. These studies linking counterfactual thinking and lying explored the determinants of closeness and exceptionality but ignored the determinant of controllability.

However, Briazu et al. (2017) pointed out that generating counterfactuals requires people to imagine alternatives. Conversely, observing desired alternatives (e.g., an additional rolled number that is larger than the actual number) allows participants to actually see a desired alternative outcome that nearly happened; as such, participants would not need to rely on counterfactual thinking about desired alternatives. To further explore the direct link between counterfactual thinking and lying, Briazu et al. (2017) conducted a series of studies using stories to prime counterfactual thinking. The stories were based on earlier work (e.g., Kahneman and Tversky, 1982; Roese and Olson, 1996) highlighting closeness and exceptionality as determinants that boosted people’s likelihood of thinking counterfactually. Briazu et al. (2017) found that participants in the high counterfactual thinking group reading stories with counterfactual determinants generated more lies than participants in the low counterfactual thinking group reading stories without such determinants. Briazu et al. (2017) asserted that this tendency reflects the close link between counterfactual thinking and lying.

## Information-Conveying Function of Speculative Thinking

Byrne (2002) suggested that people abide by certain principles to understand counterfactuals given the limitations of working memory (Johnson-Laird and Byrne, 1991). One principle is that people hold in mind only true possibilities when reading counterfactual conditionals. For example, for the counterfactual conditional “If I had come home earlier, I would have been able to get the delivery,” people represent the true possibilities of “If I had come home earlier, then I would have gotten the delivery” and “If I had not come home earlier, then I would not have gotten the delivery.” They do not hold false possibilities in mind (e.g., “If I had come home later, then I would have gotten the delivery”). Also, when reading counterfactual conditionals, people can understand what is false but could have been true (Byrne, 1997), indicating counterfactual reasoning. For instance, people would store two possibilities for the counterfactual statement “He could have chosen the usual route,” this statement suggests the presupposed fact that the protagonist did not choose his usual route. The other possibility is that the protagonist could have chosen his usual route, as this option was possible in the past.

De Vega et al. (2007) explored the difference between processing counterfactual and factual information. They asked participants to read a story about a protagonist buying a lottery ticket. The story opened with some background about the protagonist hearing lottery information on the radio, followed by either a factual or counterfactual statement. The counterfactual statement was “If Mary had won the lottery, she would have bought a Mercedes car,” the factual statement was “As Mary had won the lottery, the first thing she did was buy a Mercedes car.” Finally, participants read the story’s outcome – the protagonist either tore up the lottery ticket or sat in her car and felt like a queen. The authors measured participants’ time spent reading the story. Findings showed that when the final outcome concerned the protagonist tearing up the lottery ticket, participants spent less time reading the story with the counterfactual sentence than the story with the factual sentence. However, when the outcome entailed the protagonist sitting in her car and feeling like a queen, participants spent less time reading the story with the factual sentence than that with the counterfactual sentence. Essentially, after reading counterfactual statements, statements’ factual and counterfactual information each seems temporarily available. Counterfactual or factual information only disappears when the final outcome occurs.

Ferguson and Jayes (2018) explored factors influencing how people process counterfactuals. Specifically, they asked participants to read a counterfactual statement (written from either first- or third-person point of view) about a person preparing dinner. They also controlled the sentences’ plausibility by providing plausible, implausible, and anomalous versions of the counterfactual sentences. The study involved a 2 [point of view: first-person vs. third-person (i.e., self vs. other)] × 3 (counterfactual: plausible vs. implausible vs. anomalous) design. For example, the “other–anomalous” version of the sentence read “If Sophie had used a pump, she would have prepared the carrots in time for dinner,” the “self–plausible” version read “If you had used a knife, you would have prepared the carrots in time for dinner,” and in the “implausible” versions, the protagonist used an axe to prepare dinner. Participants’ reading time was measured, similar to in De Vega et al. (2007) study. Ferguson and Jayes (2018) found that participants displayed a shorter reading time when reading a plausible counterfactual sentence than an implausible or anomalous counterfactual sentence. No difference emerged in reading time for sentences written in first- or third-person. This implausibility effect implies that people have more difficulty processing implausible/anomalous information than plausible information.

Researchers have also explored what people understand when reading counterfactuals. Most studies have included sentence probe tasks (e.g., De Vega et al., 2007; De Vega and Urrutia, 2012) or measured participants’ reading time to discern the information people glean from counterfactuals (e.g., Santamaría et al., 2005; Ferguson and Sanford, 2008; Ferguson et al., 2008; Ferguson, 2012; Ferguson and Jayes, 2018). Several experiments (e.g., Santamaría et al., 2005; Ferguson and Sanford, 2008; Ferguson, 2012) showed that people hold various alternative possibilities in mind when processing counterfactual information compared with factual information. Individuals also generate inferences consistent with the counterfactual world.

## Excuses-Providing Function of Speculative Thinking

McCrea (2008) proposed that, because counterfactual thinking involves finding reasons for past failed attempts, such thinking can offer excuses for prior failure and poor performance. McCrea (2008) particularly suggested that counterfactual thinking may reduce one’s motivation to improve their performance in addition to reducing their actual performance on future tasks. This pattern directly contradicts other claims (e.g., Markman et al., 2008) that counterfactual thinking can enhance people’s general motivation for future tasks. For instance, Markman et al. (2008) asked participants to complete an anagram task and then instructed them to generate either upward or downward counterfactual thoughts about the task. Upward and downward counterfactual thinking were each found to bolster participants’ motivation to devote more effort to subsequent tasks. These contrasting results highlight the need for further research into the effects of controllable and uncontrollable counterfactual modifications on motivation.

## General Discussion

In this review, we have explored the functions of speculative thinking from a broader perspective than the traditional functional theory and compared functions of speculative thinking about the past and future. The functions of speculative thinking discussed in this paper generally revolve around cognitive features, while other aspects (e.g., emotion-related functions) are less addressed. Specifically, we examined several non-preparatory functions of speculative thinking that have not yet been thoroughly considered: (a) the function of conveying information and (b) the function of supporting lying. We also investigated temporal asymmetry in speculative thinking; one view focuses on psychological distance in speculative thinking about the past and future, and the other focuses on the temporal asymmetry and reality/hypothetical differences in the preparatory function of such thinking. In summary, our review indicates that a broader functional theory is needed to address non-preparatory functions and the traditional preparatory function. A broader theory should also cover all speculative thinking about the past and future rather than simply counterfactual thinking.

In essence, this review makes a different theoretical proposal based on the functional theory of counterfactual thinking (i.e., Epstude and Roese, 2008). First, this review extends the topic of interest from counterfactual thinking to general speculative thinking: our review includes counterfactual thinking, prefactual thinking, and other speculative thinking about the past and future. Second, this review extends the notion of “function” (as discussed in the functional theory) from the preparatory function in particular to conveying information, generating lies, reality/hypothetical differences in the preparatory function, and temporal asymmetry in these functions. Our review thus paints a more vivid picture of the functions of speculative thinking with respect to inferences, general speculative thinking, construal level, and deception. This discussion enriches the functional theory of counterfactual thinking.

### Future Directions

This paper synthesizes research findings regarding potential functions of speculative thinking. After combing through the literature, we can raise several questions to be addressed in subsequent work as follows (we wish to acknowledge the reviewer for their contributions to these questions). (1) Previous research on counterfactual thinking activation has focused on situational factors (e.g., exceptionality, closeness) but overlooked whether specific behavioral goals (e.g., making excuses for lies, self-improvement) can trigger speculative thinking. More importantly, if different behavioral goals can activate speculative thinking, does the content of speculative thinking sparked by behavioral goals vary? Taking counterfactual thinking as an example, the goal of avoiding blame (e.g., self-blame) may lead people to focus on situations over which they have no control, whereas the goal of self-improvement may lead people to focus on aspects over which they have control. (2) It would be interesting to explore how the content-neutral pathway of counterfactual thinking is triggered and whether it can also serve different functions. Given that the content of thoughts is unrelated to any goal or desired outcome in this case, subsequent studies can explore whether the consequences of such thoughts can be controlled or regulated in any way.

Regarding the first question, the literature has offered hints from the perspective of self-blame avoidance. Specifically, research suggests that self-blame is related to perceived control (e.g., Tennen et al., 1986; Vasconcelos e Sa et al., 2017). Tennen et al. (1986) focused on self-blame among mothers whose infants were in the hospital for serious perinatal complications, finding that the mothers’ perceived control over their infants’ medical problems and recovery was positively correlated with self-blame after discharge. Additionally, Vasconcelos e Sa et al. (2017) suggested that as the level of self-blame increases, people attribute more controllability to past events and possess a greater sense of control over similar future events. Self-blame thus inspires one’s belief that past events were controllable (i.e., could have been controlled). We therefore presume that, when thinking counterfactually to provide excuses, people may focus on an uncontrollable event to avoid self-blame. Similarly, Tennen et al. (1986) suggested that people have self-protective motives to avoid blame.

## Conclusion

In closing, this review provides a comprehensive picture of the functions of speculative thinking by detailing the functions of broad speculative thinking based on preparatory and non-preparatory functions. Our review indicates that speculative thinking serves other functions (e.g., conveying information, supporting lies) apart from preparatory functions and that differences exist between various forms of such thinking. Despite extensive research on counterfactual thinking, studies of general speculative thinking (e.g., counterfactual thinking, prefactual thinking) are rare. Future studies can further advance theories related to general speculative thinking.

## Author Contributions

LH was mainly in charge of writing and editing the manuscript. YX and XC contributed to play guiding roles. All authors contributed to the article and approved the submitted version.

## Conflict of Interest

The authors declare that the research was conducted in the absence of any commercial or financial relationships that could be construed as a potential conflict of interest.

## Publisher’s Note

All claims expressed in this article are solely those of the authors and do not necessarily represent those of their affiliated organizations, or those of the publisher, the editors and the reviewers. Any product that may be evaluated in this article, or claim that may be made by its manufacturer, is not guaranteed or endorsed by the publisher.
